# Rapid and effective removal of copper, nitrate and trichloromethane from aqueous media by aluminium alloys

**DOI:** 10.1016/j.heliyon.2023.e23422

**Published:** 2023-12-06

**Authors:** Jingqi Zhang, Ying Song, Jingbo Chao, Hai Huang, Dazhi Liu, Frederic Coulon, Xiao Jin Yang

**Affiliations:** a State Key Laboratory of Chemical Resource Engineering, Beijing University of Chemical Technology, Beijing, 100029, China; b Chemical Metrology and Analytical Science Division, National Institute of Metrology, Beijing, 100029, China; c Research & Development Centre, China State Science Dingshi Environmental Engineering Co., Ltd, Beijing, 100102, China; d Tangshan Weihao Magnesium Powder Co., Ltd, Qianan, Hebei, 064406, China; e School of Water, Energy and Environment, Cranfield University, Cranfield, MK43 0AL, UK

**Keywords:** Aluminum alloys, Nitrate, Copper, Trichloromethane, Pretreatment

## Abstract

Zero-valent iron (ZVI) has been extensively studied for its efficacy in removing heavy metals, nitrate, and chlorinated organic compounds from contaminated water. However, its limited effectiveness due to rapid passivation and poor selectivity is prompting for alternative solutions, such as the use of aluminium alloys. In this study, the efficacy of five distinct aluminium alloys, namely Al–Mg, Al–Fe, Al–Cu, and Al–Ni, each comprising 50 % Al by mass at a concentration of 10 g/L, was assessed using copper, nitrate and trichloromethane (TCM) as model contaminants. Results show that chemical pollutants reacted immediately with Al–Mg. On the contrary, the remaining three alloys exhibited a delay of 24 h before demonstrating significant reactivity. Remarkably, Al–Mg alloy reduced nitrate exclusively to ammonium, indicating minimal preference for nitrate reduction to N_2_. In contrast, the Al–Cu, Al–Ni, and Al–Fe alloys exhibited N_2_ selectivity of 3 %, 5 %, and 19 %, respectively. The removal efficiency of copper, nitrate and TCM reached 99 % within 24 h, 95 % within 48h and 48 % within 48h, respectively. Noteworthy findings included the correlation between Fe concentration within the Al–Fe alloy and an increased N_2_ selectivity from 9.3 % to 24.1 %. This resulted in an increase of Fe concentration from 10 % to 58 % albeit with a concurrent reduction in reactivity. Cu^2+^ removal by Al–Fe alloy occurred via direct electron transfer, while the removal of nitrate and TCM was facilitated by atomic hydrogen generated by the alloy's hydrolysis. Intriguingly, nitrate and TCM suppressed Cu^2+^ reduction, whereas Cu^2+^ improved nitrate reduction and TCM degradation. These findings demonstrate the great potential of Al–Mg and Al–Fe alloys as highly efficient agents for water remediation.

## 1 Introduction

Water pollution caused by heavy metals, nitrate, and chlorinated hydrocarbons such as trichloromethane, pose serious threats to both human health and the ecological environment [[1], [2], [3], [4], [5], [6]]. To mitigate this issue, a variety of techniques have been employed for removing these pollutants from contaminated water, including but not limited to adsorption, anion exchange, membrane filtration, biological methods, and chemical reduction [1,2,[7], [8], [9], [10], [11], [12]]. Among these various methods, chemical reduction using zero-valent iron (ZVI) and zero-valent aluminum (ZVAl), and bimetallic systems such as Fe/Al, Fe/Ni, Pd/Al has been found to be a cost-effective and efficient method for removing multiple contaminants [9,[13], [14], [15], [16], [17], [18]].

Nonetheless, it is worth reporting that both ZVI and ZVAl undergo rapid passivation [19,20]. Both materials were found to exclusively reduce nitrate to ammonium [15,21]. However, the primary goal in addressing nitrate-contaminated water is the conversion of nitrate to N_2_, as ammonium poses a significantly greater risk to aquatic organisms compared to nitrate. Additionally, conventional bimetals are not uniform and are susceptible the detachment of the two metals which compromise their galvanic and catalytic effects [20,[22], [23], [24]]. Bimetallic alloys avoid these problems of conventional bimetals [25] and Al–Fe and Al–Ni alloys have been successfully used to remove heavy metals, nitrate and/or chlorinated organics from water [1,21,24,[26], [27], [28], [29], [30], [31], [32], [33]]. Notably, it was observed that the Al–Fe alloy exhibited high efficiency in converting nitrate, achieving a conversion rate from 10 to 60 % across a broad range from pH 2 to 12. The intermetallic Al_13_Fe_4_ in the Al–Fe alloy was identified as the catalyst enhancing the efficiency and N_2_ selectivity [21]. The intermetallic Al_12_Mg_17_ within the Al–Mg alloy was also noted for its ability to catalyze the reduction of Ni^2+^ by Mg [34]. Further to this, it was also shown that Al–Ni alloy was able to catalyze the hydrodechlorination of 2-chlorophenol [26,29].

The presence of Cu^2+^ was found to enhance the nitrate reduction to N_2_ when using Al–Fe alloys. This enhancement occurred due to the increasing concentration of nitrite intermediates, which facilitated the oxidation of ammonium to N_2_. Additionally, Cu^2+^ was also seen as a factor that promoted the reduction of nitrite to ammonium [15,30]. To date, our understanding of the catalytic effects arising from the intermetallic compounds within Al alloys and the presence of heavy metals, whether alloyed with Al or existing in the contaminated water, as well as their interactions with various contaminants in water, remain scarce. The primary objective of this study is to conduct a comprehensive investigation into the efficacy of Al–Mg, Al–Fe, Al–Ni, and Al–Cu alloys in the decontamination of water. The study focuses on using copper, nitrate, and trichloromethane as representative model pollutants to understand how these alloys perform. Additionally, the study aims to elucidate the intricate interactions between these pollutants and the alloys. Furthermore, it seeks to explore the potential applications of these alloys in treating acid mine drainage (AMD) originating from iron and copper mining sites.

## 2 Materials and methods

### 2.1 Chemicals, materials and acid mine drainage

Analytical grade chemicals were used throughout the study. CuCl_2_, Cu(NO_3_)_2_, KNO_3_ and TCM purchased from Beijing Chemicals, China. The initial concentrations of Cu^2+^, NO_3_^−^ and TCM in spiked water samples were 5 mg/L, 20 mg-N/L and 120–153 μg/L, respectively. The spiked water samples were prepared by dissolving CuCl_2_, Cu(NO_3_)_2_, KNO_3_ and TCM in deionized water. The Al alloys were composed of 50 % of the second metal for Al–Mg, Al–Ni, and Al–Cu alloys (referred to as Al–Mg50, Al–Ni50, and Al–Cu50). Additionally, three variations of Al–Fe alloys, termed Al–Fe10, Al–Fe50, and Al–Fe58, were also evaluated. The particle size for all of the alloys ranged between 100-and 200 meshes. Al–Mg50 was provided by Tangshan Weihao Magnesium Powder Co., Ltd, Qianan, Hebei, China, Al–Fe alloys were purchased from Hubei Tianhui Special Metal Materials Co., Ltd (Hanchuan, China), Al–Cu50 alloy was obtained from Jiangsu Youlian Metals Co., Ltd (Nantong, China) and Al–Ni50 alloy (325 meshes, 45 μm) was obtained from Aladdin reagent (Shanghai) Co., Ltd. Samples of acid mine drainage (AMD) were taken from mining pits from South China (Iron ores 118.5°E, 31.7°N and copper ores 117.7°E, 29.0°N).

### 2.2 Alloy pretreatment and pollutant removal

The alloy powders (5 g) were soaked in 500 mL deionized water for pretreatment and hydrogen was collected into a measuring cylinder by water drainage. After pretreatment, alloy powders were collected and dried at 40 °C in a vacuum oven for 12 h. Then, 5 g pretreated alloy powders were added to 500 mL contaminated water samples and the mixture was stirred at 900 rpm at 25 °C. At given time intervals, 5 mL of sample were withdrawn and filtered through a 0.22 μm membrane filter for pH measurement and analysis of nitrate, nitrite, metals, and TCM concentrations. The experiments were performed in duplicates and the average removal rate was calculated according to equation 1

Eq. 1 $$removal\;rate=\frac{C-C_{0}}{C_{0}}\times 100\%$$

### 2.3 Analytical methods and material characterization

The pH of the solution was measured using a Mettler Toledo, FE 20 pH meter. The concentrations of nitrate and nitrite were determined by ion chromatography (Dionex, ICS-900, USA) using a solution containing 4.5 mM Na_2_CO_3_ and 0.8 mM NaHCO_3_. Ammonium concentration was quantified by optical absorption method using a UV–vis spectroscopy (Persee, TU-1900, China) at a wavelength of 697 nm, where salicylic acid and hypochlorous acid was used as the coloring reagents [35]. The selectivity of nitrate reduction to N_2_ was estimated by mass balance of total nitrogen, NO_3_^−^-N, NO_2_^−^-N and NH_4_^+^-N as previously described [15,21].

The determination of TCM was carried out using GC (GC-2010 plus, Shimadzu, Japan) equipped with an electron capture detector [31]. Concentration of metals was determined by induced coupled plasma mass spectrometry ICP-MS (7500, Agilent, America) and ICP-OES (iCAP 6000, America). The morphology and chemical composition of the alloys were characterized by scanning electron microscopy (Hitachi, S4800-SEM, Japan) and X-ray diffraction (Bruker, D8 Advance, Germany) with a scanning range from 15° to 90° and a scanning speed of 5° min^−1^.

## 3 Result and discussion

### 3.1 Effect of alloying elements

**Fig. 1:**
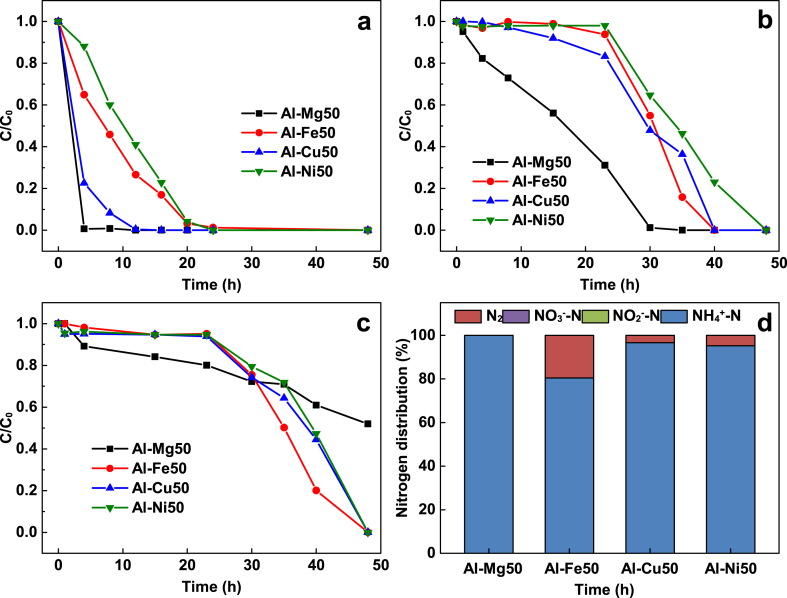
Removal of Cu, nitrate and TCM from water (**a**) Cu(II), (**b**) NO_3_^−^-N, (**c**) TCM and (**d**) nitrogen distribution by Al-based alloys. Al alloy 5 g, volume of solution 500 mL, temperature 25 ± 1 °C, particle size 150 μm, initial concentration: [NO_3_^−^-N] = 22.7 mg-N/L, [Cu(II)] = 5 mg/L, [TCM] = 153 μg/L; initial pH 6.3 ± 0.2. The nitrogen distribution of NO_3_^−^-N and NO_2_^−^-N are below 1 % in Fig. 1d.

The removal rate of Cu^2+^, NO_3_^−^ and TCM and the selectivity of NO_3_^−^ reduction by Al–Mg, Al–Fe, Al–Cu and Al–Ni are shown in Fig. 1. To achieve 97 % removal efficiency for Cu^2+^, NO_3_^−^, and TCM requires between 4 and 20 h, 30 and 48 h, and more than 48 h, respectively(Fig. 1a–c). Al–Mg has the highest removal rate for Cu and NO_3_^−^ but the lowest rate for TCM. The removal efficiency follows the order of Al–Mg > Al–Cu > Al–Fe > Al–Ni for Cu^2+^, Al–Mg > Al–Fe ≥ Al–Cu > Al–Ni for NO_3_^−^ and Al–Fe > Al–Cu ≥ Al–Ni > Al–Mg for TCM.

A retardation period of approximately 24 h was observed for Al–Fe, Al–Cu and Al–Ni in removing NO_3_^−^ and TCM. However, this delay was not observed for Cu^2+^. In contrast, Al–Mg alloy reacted immediately with all pollutants. The retardation effect was also observed on Al–Fe alloy for nitrate removal [32] and ZVAl for Cr(VI) removal [36]. The retardation is attributed to the protection of metal oxide layer on the surface of the alloy particles. The length of retardation can be significantly shortened by increasing the temperature of the solution [37]. It is not clear why the retardation period was not observed for Cu^2+^ removal. A plausible explanation is that Cu^2+^ can easily penetrate the thin metal oxide layer and undergo direct electron transfer with Al, while the reduction of NO_3_^−^ and TCM requires atomic hydrogen, which is produced by the reaction of Al with water molecules.

**Fig. 2:**
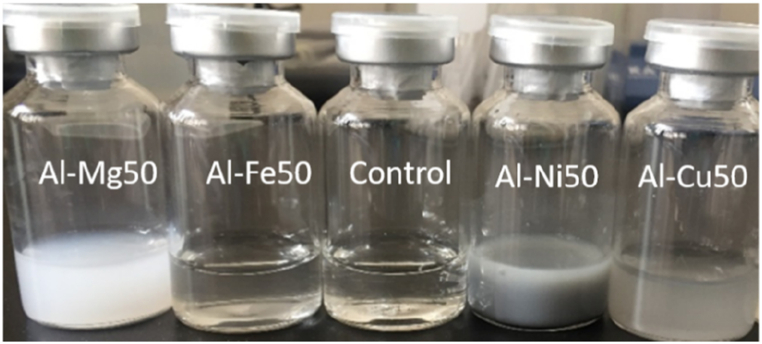
Photos of end solutions after simultaneous removal of nitrate (22.7 mg-N/L), TCM (153 μg/L) and Cu (II) (5 mg/L) by four Al-based alloys. Experimental conditions are the same as those of Fig. 1.

After the retardation period, the decrease in pollutant concentration over time follows a pseudo-zero-order reaction. The removal kinetics do not adhere to the typical rules of physico-chemical adsorption. The dominant mechanism for removing nitrate and chlorinated organic solvents, such as CHCl_3_, CCl_4_, trichloroethylene, 1, 2-dichloroethane, 2-chloro-2-methylpropane, and 1-chlorobutane, from water using ZVI, ZVAl, and Al–Fe alloys involves chemical reduction, which results in the release of Fe and Al ions [1,15,38,39]. ZVI removes Cu^2+^ through a combination of chemical reduction (Cu^2+^ → Cu^0^) and coprecipitation of copper and iron hydroxides [40]. Similarly, Al–Mg, Al–Fe, Al–Cu, and Al–Ni alloys remove Cu^2+^ through a combination of chemical reduction and coprecipitation of aluminum and copper hydroxides. The initial pH of the solution was 6.3 ± 0.2 (Fig. 1), while the final pH of the solution was ranging between 8.78 and 9.67 (Table 1) which meets the requirement for general water quality (pH 6–9) [41]. As a result of the removal process, the formation of white or grey precipitates was observed (Fig. 2). Furthermore, high concentrations of Al (5 mg/L by Al–Ni, 35 mg/L by Al–Mg, and 180 mg/L by Al–Cu) were also recorded in the solution (Table 1). Clearly, Al acts as the electron donor in these alloys for chemical reduction of pollutants, while the alloying metals (Mg, Fe, Ni, and Cu) are well-protected.

**Table 1:** Ion concentrations and final pH values in the experiments of Fig. 1.

Parameter	Al–Mg50	Al–Fe50	Al–Ni50	Al–Cu50
Al concentration (mg/L)	Al: 35	Al: 0.19	Al: 5.0	Al: 180
Elements concentration (mg/L)	Mg: 0.08	Fe: 0.05	Ni: 0.01	Cu: <0.01
Final pH	9.52	8.19	8.78	9.67

It is interesting to note that Mg is protected by Al in the Al–Mg alloy, as Mg is more active than Al (E^Ɵ^(Mg^2+^/Mg) = −2.37 V, E^Ɵ^(Al^3+^/Al) = −1.67 V). Nitrate reduction by hydrogen occurs through the pathway of N(+V) → N(+IV) → N(0) → N(-III) leading to the production of nitrite, nitrogen gas (N_2_) and ammonium [1]. Ammonium is the sole end-product of nitrate reduction by ZVI [1]. In contrast, Pd–Cu bimetal catalyzed 86 % reduction of nitrate to N_2_ [4]. Further to this, Shi's study demonstrated that Fe–Pd bimetallic nanoparticles reduced 71 % of nitrate to N_2_ at pH 8.67 and 25 °C [33].

**Fig. 3:**
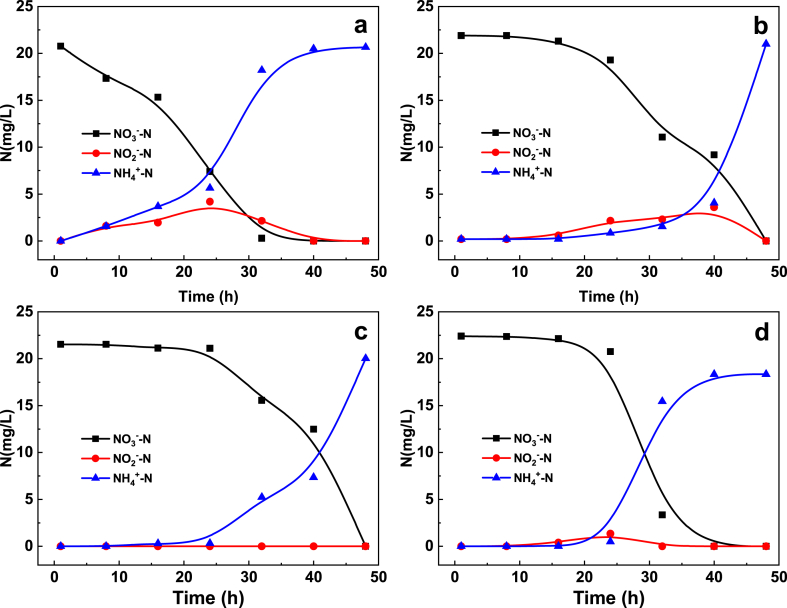
Concentration of nitrate reduction end-products during simultaneous removal of nitrate (22.7 mg-N/L), TCM (153 μg/L) and Cu (II) (5 mg/L) by (**a**) Al–Mg50, (**b**) Al–Cu50, (**c**) Al–Ni50, (**d**) Al–Fe50. Experimental conditions are the same as those of Fig. 1.

Cu and Ni have been reported to catalyze nitrate reduction by H_2_ [4,42]. By contrast, no improvements in nitrate reduction were observed using Al–Cu and Al–Ni alloys, as compared to Al–Mg and Al–Fe alloys. All four alloys produced ammonium as the major end-product, but Al–Fe alloy exhibited the highest N_2_ selectivity (Fig. 1d). Nitrite (NO_2_^−^) is known to be an intermediate product of nitrate reduction by ZVI and Al–Fe alloy [1,15,21] and is believed to enhance N_2_ selectivity [43]. Although noticeable concentrations of nitrite were detected during nitrate reduction by Al–Mg and Al–Cu (Fig. 3), there was no improvement in N_2_ selectivity. Overall, Al–Fe alloy performed the best in terms of removal efficiency, N_2_ selectivity, and metal leaching for the removal of Cu, nitrate, and TCM from water.

### 3.2 Effect of Fe content in Al–Fe alloy

**Fig. 4:**
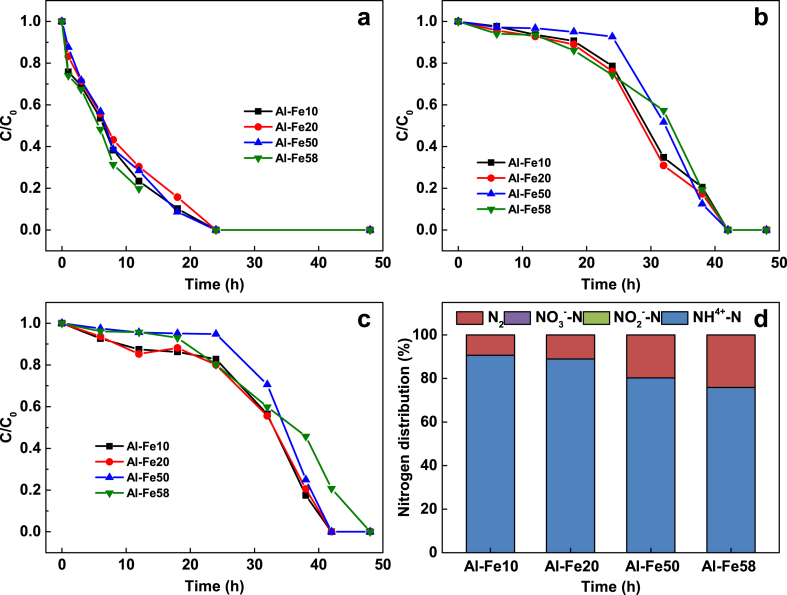
Removal of Cu, nitrate and TCM from water (**a**) Cu(II), (**b**) NO_3_^−^-N, (**c**) TCM and (**d**) nitrogen distribution by Al–Fe alloys. Volume of solution 500 mL, reduction temperature 25 ± 1 °C, particles size 150 μm, Al alloy particle loaded 5 g, initial concentration: [NO_3_^−^-N] = 22.7 mg-N/L, [Cu(II)] = 5 mg/L, [TCM] = 153 μg/L; initial 6.3 ± 0.2. The nitrogen distribution of NO_3_^−^-N and NO_2_^−^-N are below 1 % in Fig. 4d.

The effectiveness of Al–Fe alloys with varying Fe concentrations (10 %, 20 %, 50 %, and 58 %) in removing mixtures of Cu^2+^, NO_3_^−^, and TCM pollutants, as well as the nitrogen speciation, is shown in Fig. 4. The complete removal of 5 mg/L Cu^2+^, 23 mg-N/L nitrate, and 153 μg/L TCM was achieved within 24, 42, and 48 h, respectively (Fig. 4a–c). Interestingly, the N_2_ selectivity improved as the Fe concentration of Al–Fe alloy increased, with Al–Fe58 exhibiting the highest N_2_ selectivity of 24.1 % compared to Al–Fe10 alloy's 9.3 % (Fig. 4d). This trend has been observed previously and it was considered that the intermetallic Al–Fe compound Al_13_Fe_4_ catalyzes nitrate reduction to N_2_ [15,21].

### 3.3 Effect of pretreatment with deionized water

It is widely acknowledged that the reason for the retardation period in Al alloys and ZVAl is the protective metal oxide layer on their surface, as reported by Zhang [36]. To activate ZVI and ZVAl, an acid wash pretreatment is typically used to eliminate the metal oxides on the surface, as demonstrated by Han [44] and Yang [22]. On the other hand, Bao [15] showed that a deionized water soaking pretreatment for 2 h at 45 °C was effective in activating Al–Fe15 alloy. Once the metal oxide layer is removed, the fresh metal surface reacts with water, generating hydrogen and metal hydroxide. Consequently, two competing reactions, surface re-passivation and hydrolysis reaction (i.e., corrosion), may occur. Therefore, the efficiency of pollutant removal may vary depending on the duration of pretreatment.

**Fig. 5:**
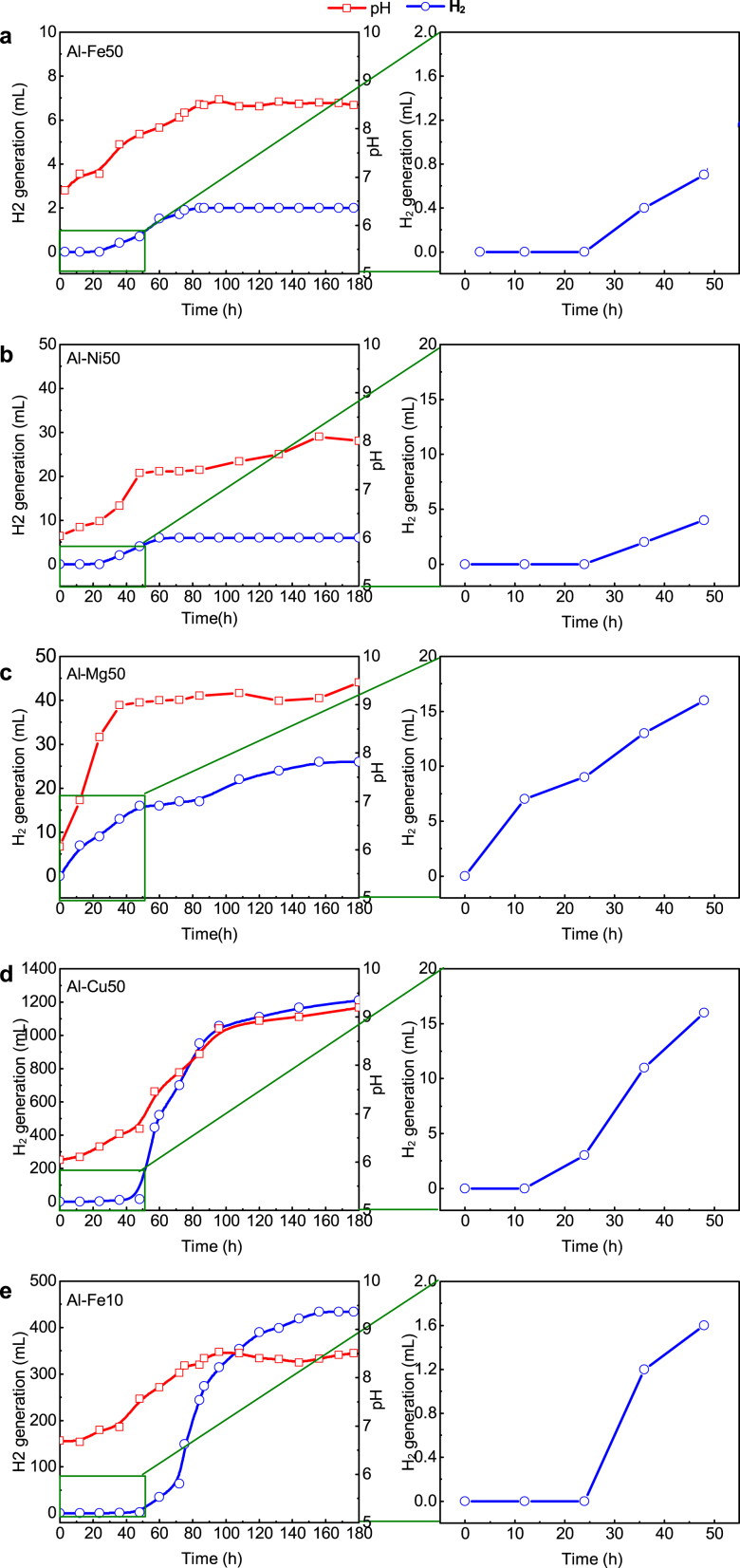
H_2_ generation and pH during Al alloys pretreatment with water. **(a)** Al–Fe50, **(b)**Al–Ni50, **(c)**Al–Mg50, **(d)**Al–Cu50 and **(e)**Al–Fe10. Solution volume 500 mL; reaction temperature 25 ± 1 °C; amount of materials 5 g.

In this study, deionized water soaking pretreatment at ambient temperature was applied to Al–Fe50, Al–Ni50, Al–Mg50, Al–Cu50, and Al–Fe10, and the hydrogen generation and pH variation during the pretreatment were measured (Fig. 5). The amount of hydrogen generated is a direct measurement of the degree of metal corrosion in aqueous solutions. The results show that the amount of hydrogen generated by Al–Fe50 and Al–Ni50 was negligible during the 180-h soaking, whereas large amounts of hydrogen were produced by Al–Mg50, Al–Cu50, and Al–Fe10. Hydrogen production by Al–Mg50 was immediate, while a retardation period of 12 and 24 h was observed for Al–Cu50 and Al–Fe10, respectively. This phenomenon may explain the removal behaviour observed in Fig. 1. Hydrogen generation by Al–Fe10 increased rapidly after 48 h of soaking and slowed down after 160 h.

**Fig. 6:**
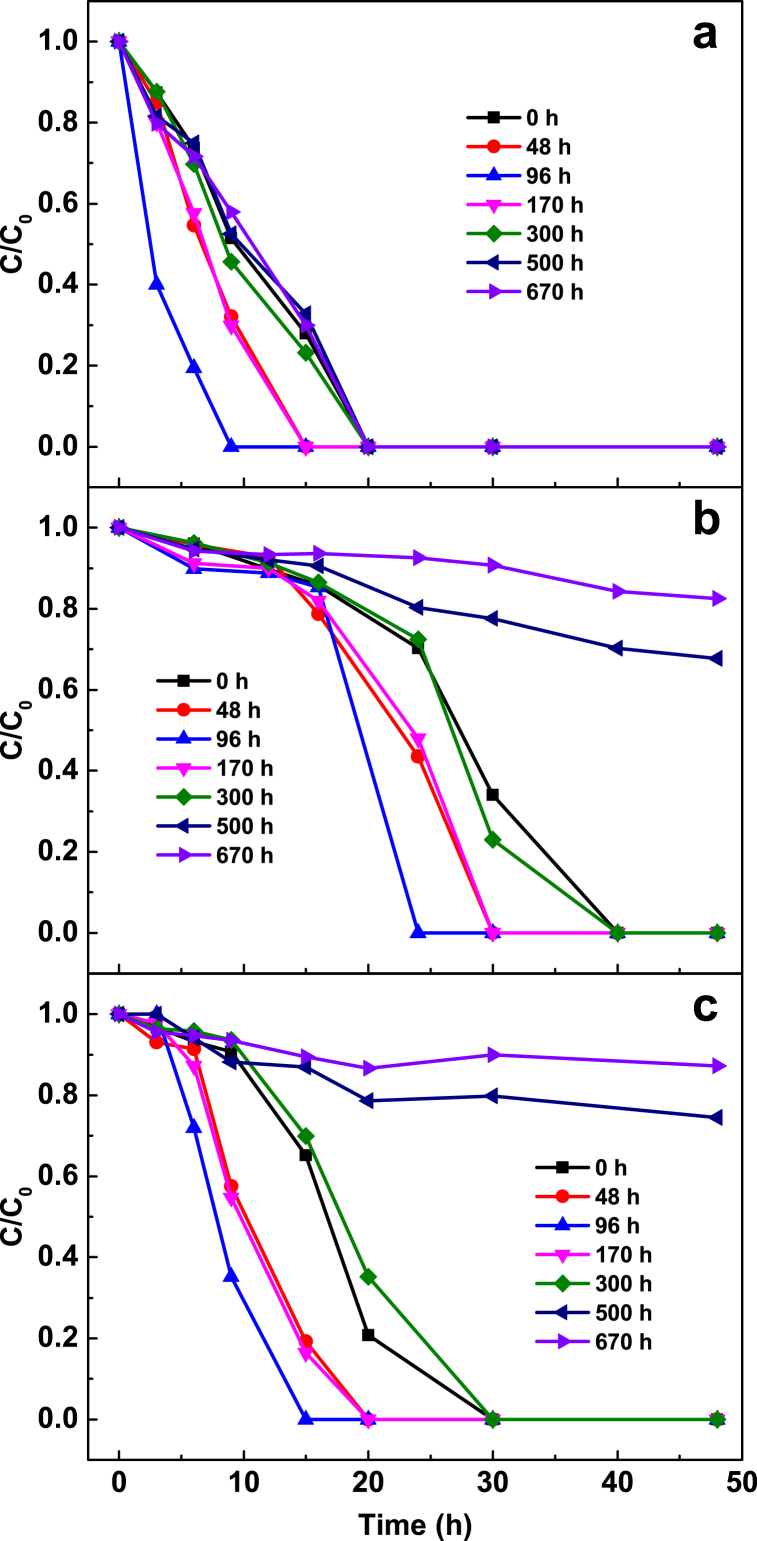
Effect of water pre-treatment time on removal of (**a**) Cu(II), (**b**) NO_3_^−^-N, (**c**) TCM by Al–Fe10 alloy particles. Solution volume 500 mL; temperature 25 ± 1 °C; particle size 100 meshes; Al–Fe10 alloy loaded 5 g; initial concentration: [NO_3_^−^-N] = 20 mg/L, [Cu(II)] = 5 mg/L, [TCM] = 120 μg/L; initial pH 6.3 ± 0.2.

**Fig. 7:**
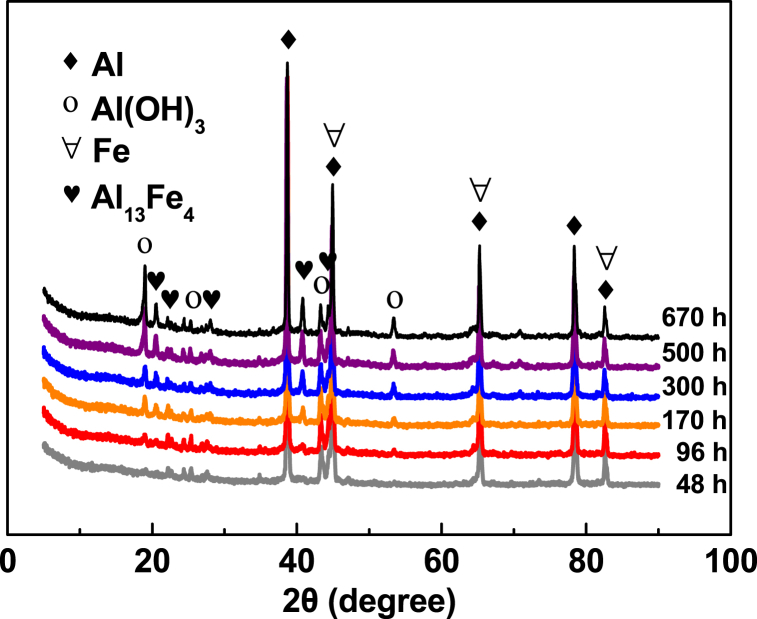
XRD patterns of Al–Fe10 alloy particles with different pretreatment time. Solution volume 500 mL; reaction temperature 25 ± 1 °C; amount of materials 5 g.

**Fig. 8:**
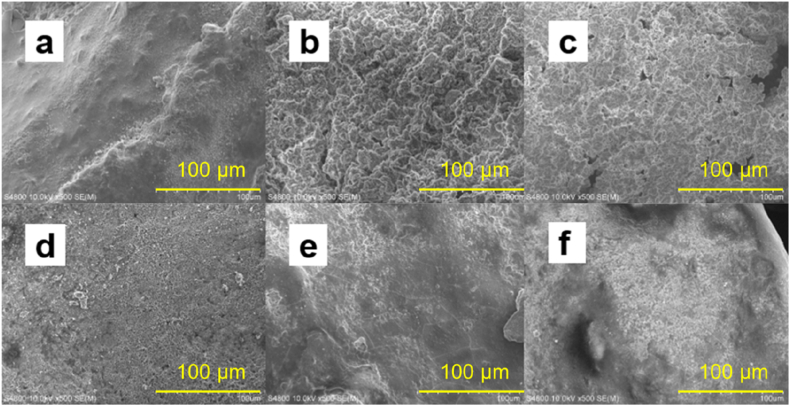
SEM images of Al–Fe10 alloy particles with different pretreatment time: (**a**) 48 h, (**b**) 96 h, (**c**) 170 h, (**d**) 300 h, (**e**) 500 h, (**f**) 670 h. Solution volume 500 mL; reaction temperature 25 ± 1 °C; amount of materials 5 g.

The effect of pretreatment time (0–670 h) on removal of Cu^2+^, NO_3_^−^ and TCM by Al–Fe10 is shown in Fig. 6. The results show that increasing the pretreatment time up to 96 h leads to an increase in removal rates, followed by a decline thereafter. The rates of pollutant removal and hydrogen generation were positively correlated, indicating that the fastest removal of pollutants coincides with the highest rate of hydrogen generation. The impact of the pretreatment time on Cu^2+^ removal is less significant than on nitrate and TCM. The difference in Cu^2+^ removal rates between 300, 500 h, and 670 h was negligible (Fig. 6a), whereas a notable difference was observed for nitrate and TCM (Fig. 6b and c). After 170 h of soaking, hydrogen generation almost stopped, while the removal rates of nitrate and TCM decreased as the pretreatment time increased. This finding supports the notion that Cu^2+^ is removed by chemical reduction via direct electron transfer from Al, whereas nitrate and TCM are removed by reaction with hydrogen. Increasing pretreatment time produced more Al(OH)_3_ (Fig. 7) and porous, rougher surface (Fig. 8). The increased surface porosity observed in alloys pretreated for 96–170 h is likely caused by H_2_ generation. In contrast, the decrease in surface porosity of alloys pretreated for 300–670 h is attributed to surface re-passivation once H_2_ generation stopped which has been also reported for ZVAl surfaces [24,45].

### 3.4 Interaction of pollutants

**Fig. 9:**
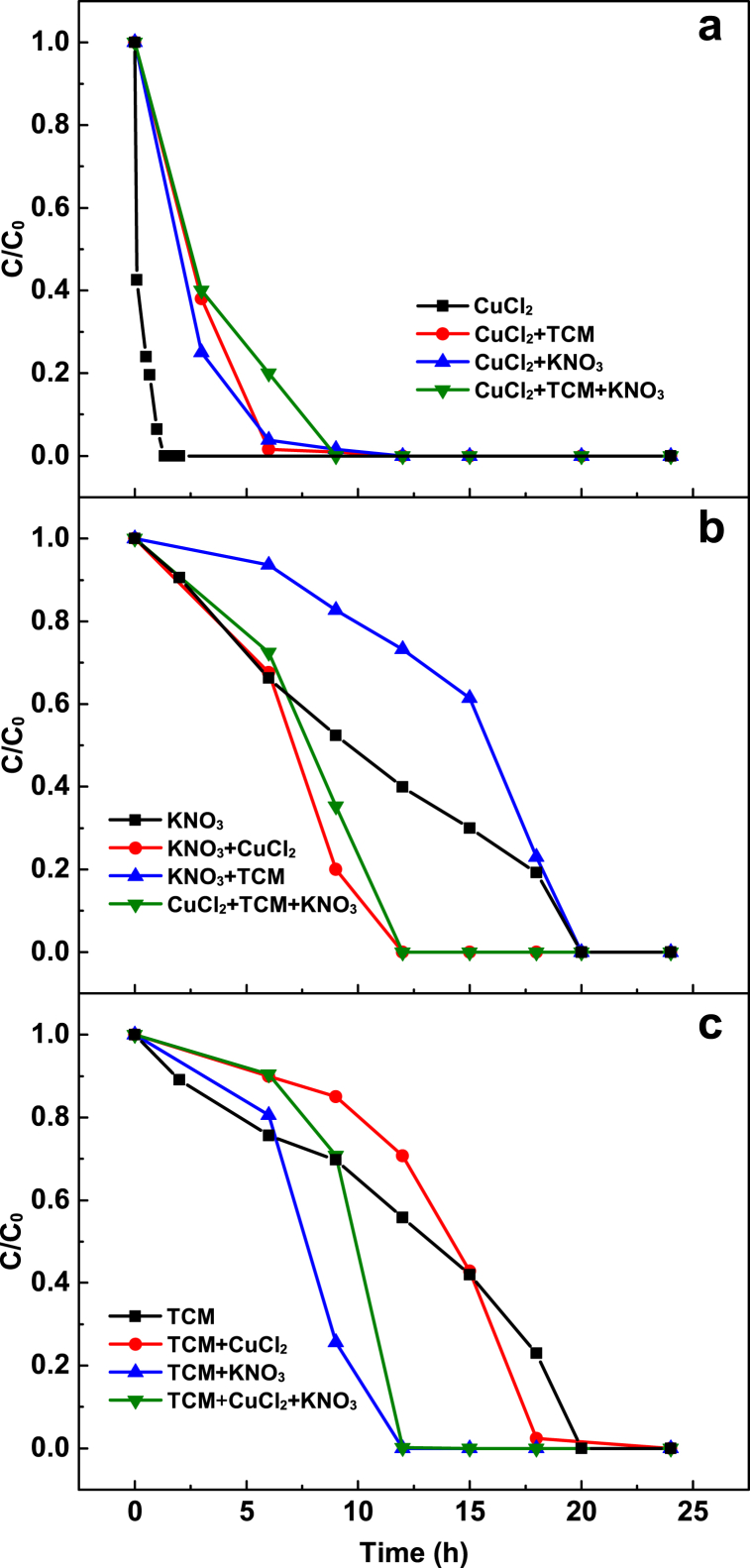
Removal kinetics of single and mixed solutions by Al–Fe10 alloy particles after pretreatment of 90 h: (**a**) Cu(II), (**b**) NO_3_^−^-N and (**c**) TCM. Solution volume 500 mL; temperature 25 ± 0.5 °C; Al–Fe10 alloy loaded 5 g; initial concentration: [NO_3_^−^-N] = 20 mg/L, [Cu(II)] = 5 mg/L, [TCM] = 120 μg/L; initial pH 6.3 ± 0.2.

Suppression and enhancement within complex mixtures of contaminants are a common phenomenon during their interactions with ZVI and Al–Fe alloys [1,32,46]. For instance, the reduction of nitrate by ZVI was hindered by trichloroethylene while it was improved in presence of Cu^2+^ [1,46]. In contrast, nitrate hindered the removal of Cu^2+^ by Al–Fe alloys [32]. In this study, nitrate and TCM significantly inhibited the removal of Cu^2+^ by Al–Fe10 (Fig. 9a). Conversely, the presence of Cu^2+^ enhanced the removal of nitrate and TCM by Al–Fe10 (Fig. 9b and c). After 6 h of reaction, 97 % of Cu^2+^ was reduced to Cu^0^ while the removal of nitrate and TCM was improved. TCM hindered the reduction of nitrate, whereas nitrate enhanced TCM removal.

### 3.5 Treatment of AMD

Al–Fe50 and Al–Mg50 alloys were applied to treat AMD samples from iron and copper ore mines (Table 2). For these two typical samples, Al–Mg50 was found to be more efficient than Al–Fe50 for removing heavy metals and raising the pH of the treated samples. Specifically, Al–Mg50 removes >98 % of Cu, Ni, Zn, Fe and Al and >63.2 % of Mn and Cr, while raising the acidic pH to neutral pH. Moreover, the concentration of Al ions was reduced to <0.1 mg/L from 550 to 730 mg/L. In contrast, Al–Fe50 was effective to remove Cu and poor to other heavy metals (Cr, Fe, Mn, Ni, Zn and Al) while producing secondary contamination by Al and Fe. The pH of both samples was slightly raised to 3.7–3.9, indicating that the Al–Fe alloy was re-passivated.

**Table 2:** Treatment of acid mine drainage (AMD) by Al–Fe50 and Al–Mg50.

Water quality indicator	Acid mine drainage #1 (Iron ore mine, 118.5°E, 31.7°N)				Acid mine drainage #2 (copper ore mine, 117.7°E, 29.0°N)			
	Before		After treatment		Before		After treatment	
	treatment		Al–Fe50	Al–Mg50	treatment		Al–Fe50	Al–Mg50
pH	3.08		3.93	7.35	2.47		3.69	7.45
Cu	24.64		0.30	<0.05	88.5		0.15	<0.05
Ni	1.96		2.33	<0.05	4.34		4.82	<0.05
Zn	9.46		9.41	<0.05	6.14		5.60	<0.05
Fe	49.4		268.6	0.59	544.5		863	0.58
Mn	276.4		280.6	84.4	122		115.5	10.96
Cr	0.07		0.58	<0.05	0.51		0.72	<0.05
Ca	438.3		410.6	373.5	441.2		395.3	347.6
Al	553.7		872.5	<0.1	731.4		865.5	<0.1
Mg	1608.0		1299.5	1610.5	1503		1146.5	1694
K	26.40		5.26	5.09	30.69		2.88	2.27

Note: The concentration of metals is in mg/L. AMD volume, 50 mL; temperature 16 ± 0.5 °C; alloy particle size 100 meshes; alloy loaded 0.5 g.

Limestone and lime are commonly used for AMD treatment, but they have many drawbacks, such as the difficulties in maintaining neutral pH, the formation of calcium sulfate suspensions and the disposal of large volumes of sludges [[47], [48], [49]]. Although Al–Mg alloy is much more expensive than lime, the cost of AMD treatment using Al–Mg alloy can be reduced by using scraps from Al–Mg alloy industries and producing layered double hydroxides (LDH), which has widespread applications in industrial catalysis, soil and water remediation [[50], [51], [52], [53]].

## 4 Conclusion

Among aluminum alloys (Al–Fe50, Al–Mg50, Al–Ni50 and Al–Cu50) for removing Cu^2+^, nitrate and trichloromethane from contaminated water and treating acid mine drainage, Al–Fe alloy demonstrated the best efficiency, selectivity, and minimal secondary contamination at near neutral pH, while Al–Mg alloy was the most effective to treat acidic wastewater. A simple soaking pretreatment was found to activate Al alloys, and Cu^2+^ removal occurred via direct electron transfer while nitrate and trichloromethane were removed by atomic hydrogen generated by alloy's hydrolysis. Nitrate and trichloromethane suppressed Cu^2+^ reduction, while Cu^2+^ improved the rate of nitrate and trichloromethane removal. Al–Mg alloy was effective to maintain neutral pH and simultaneously remove heavy metals and other pollutants without the use of additional chemicals. Further investigation into detailed mechanisms of complex mixtures of heavy metals, anions and organic pollutants by Al–Mg alloys is required to explore its applications to environmental remediation. Overall, Al–Fe and Al–Mg alloys are promising options for treating a wide variety of wastewater.

## Data availability statement

All data are included in the article and the supplementary material.

## Ethics statement

Review and/or approval by an ethics committee was not needed for this study because the study did not involve ethical considerations.

## CRediT authorship contribution statement

**Jingqi Zhang:** Writing – original draft, Formal analysis. **Ying Song:** Data curation. **Jingbo Chao:** Resources. **Hai Huang:** Resources. **Dazhi Liu:** Resources. **Frederic Coulon:** Writing – review & editing. **Xiao Jin Yang:** Writing – review & editing, Conceptualization.

## Declaration of competing interest

The authors declare the following financial interests/personal relationships which may be considered as potential competing interests:Frederic Coulon reports a relationship with Heliyon that includes: employment.
